# Elevated Soluble Triggering Receptor Expressed on Myeloid Cells (sTREM)-1 Levels in Maternal Serum during Term and Preterm Labor

**DOI:** 10.1371/journal.pone.0056050

**Published:** 2013-02-28

**Authors:** Inge Tency, Hans Verstraelen, Bart Saerens, Bruno Verhasselt, Mario Vaneechoutte, Olivier Degomme, Rita Verhelst, Marleen Temmerman

**Affiliations:** 1 Department of Obstetrics and Gynecology, Faculty of Medicine and Health Sciences, Ghent University, Ghent, Belgium; 2 Department of Clinical Chemistry, Microbiology & Immunology, Faculty of Medicine and Health Sciences, Ghent University, Ghent, Belgium; 3 International Centre for Reproductive Health, Department of Obstetrics and Gynecology, Faculty of Medicine and Health Sciences, Ghent University, Ghent, Belgium; University of Cape Town, South Africa

## Abstract

**Background:**

Infection and inflammation are important mechanisms leading to preterm birth. Soluble triggering receptor expressed on myeloid cells-1 (sTREM-1) belongs to a family of cell surface receptors that seems to play an important role in fine-tuning the immune response. It has been demonstrated that sTREM-1 is involved in bacterial infection as well as in non-infectious inflammatory conditions. Few studies have investigated serum sTREM-1 expression during preterm labor. Therefore, the purpose of this study was to assess sTREM-1 concentrations in maternal serum during term and preterm labor.

**Methods:**

This case control study included 176 singleton pregnancies in the following groups: patients in (1) preterm labor, delivered before 34 weeks (*PTB*) (n = 52); (2) *GA matched controls*, not in labor, matched for gestational age (GA) with the PTB group (n = 52); (3) *at term in labor* (n = 40) and (4) *at term not in labor* (n = 32). sTREM-1 concentrations were determined by enzyme-linked immunoassay.

**Results:**

sTREM-1 was detected in all serum samples. Median sTREM-1 concentrations were significantly higher in women with *PTB* vs. *GA matched controls* (367 pg/ml, interquartile range (IQR) 304–483 vs. 273 pg/ml, IQR 208–334; P<0.001) and in women *at term in labor* vs. *at term not in labor* (300 pg/ml, IQR 239–353 vs. 228 pg/ml, IQR 174–285; P<0.001). Women with *PTB* had significantly higher levels of sTREM-1 compared to women *at term in labor* (P = 0.004). Multiple regression analysis, with groups recoded as three key covariates (labor, preterm and rupture of the membranes), showed significantly higher sTREM-1 concentrations for labor (+30%, P<0.001) and preterm (+15%, P = 0.005) after adjusting for educational level, history of PTB and sample age.

**Conclusions:**

sTREM-1 concentrations in maternal serum were elevated during spontaneous term and preterm labor and sTREM-1 levels were significantly higher in preterm labor.

## Introduction

Preterm birth (PTB), defined as a delivery before 37 completed weeks of gestation, remains a key issue in modern obstetrics. PTB is the major cause of neonatal morbidity and mortality in developed countries [1]. Infection and inflammation are important mechanisms leading to PTB [2], [3]. Intra-uterine infection caused by bacteria is considered to be the primary cause of PTB [4]–[7] and presumably evokes an immune response that involves the release of cytokines and chemokines, prostaglandins and matrix-degrading enzymes. These substances trigger uterine contractions, membrane rupture and cervical ripening [4]. Evidence suggests that most intra-uterine infections are chronic and subclinical in nature and consequently hard to diagnose before labor or rupture of membranes [1], [4], [5]. Therefore a diagnostic marker of subclinical infection or inflammation would be most useful to identify women at risk for PTB.

Recently, a family of cell surface receptors, the triggering receptor expressed on myeloid cells (TREM) proteins, has been discovered that seems to play an important role in fine-tuning the innate immune response during infectious diseases. TREM-1 is a transmembrane glycoprotein, mainly expressed in monocytes and neutrophils [8]–[14]. Studies have shown that TREM-1 expression is upregulated in response to lipopolysaccharide (LPS) and other microbial components [8]–[14]. TREM-1 synergizes with pathogen recognition receptors, including Toll-like receptors (TLRs), which in turn, increase cytokine production (e.g. IL-8, TNF-α and IL-1α)[8]–[14]. Hence, TREM-1 functions as an amplifier of the inflammatory response in the context of bacterial infection [8]–[14]. During infection, soluble TREM-1 (sTREM-1) is generated through proteolytic cleavage of the TREM-1 ectodomain by matrix metalloproteinases [10], [14]. sTREM-1 is detectable in various body fluids [10], [12] and has been detected in plasma, broncho-alveolar lavage fluid, pleural fluid, cerebrospinal fluid and urine from intensive care patients with bacterial infections [12]. Recent studies have demonstrated that TREM-1 is also involved in non-infectious inflammatory conditions such as rheumatoid arthritis [15] and inflammatory bowel disease [16].

Few studies have investigated the role of sTREM-1 during PTB [6], [17]–[19]. Menon et al [18] demonstrated that both lipopolysaccharide (LPS) and preterm labor induced fetal membrane TREM-1 expression. In the presence of intra-uterine infection, amniotic fluid sTREM-1 concentrations were significantly higher in patients with preterm labour (PTL) [6], [18] or preterm premature rupture of the membranes (PPROM) [6]. However, amniotic fluid TREM-levels did not predict PTB within 7 days in women with PPROM [20]. Elevated serum concentrations of sTREM-1 in the second trimester were associated with an increased risk of PTB in asymptomatic high risk patients [19], but not in low-risk women [17]. Furthermore, it has been shown that amniotic fluid concentration of sTREM-1 increases with advancing gestational age [6]. To our knowledge, only two studies [21], [22] evaluated serum sTREM-1 concentrations in patients with preterm labor. Blood sampling has the advantage of being less invasive than amniocentesis and is therefore a more appropriate method to perform in pregnancy. However, these studies did not include women with term labor. There is accumulating evidence that inflammation has also been implicated in the mechanism of spontaneous term parturition [2], [7]. Youssef et al [23] demonstrated that TREM-1 is expressed in myometrium and cervix at term and found that sTREM-1 is upregulated in both tissues with the onset of labor.

Therefore, the objective of the present study was to evaluate whether sTREM-1 is upregulated in maternal serum during term and preterm labor vs. non laboring controls and to assess a possible relationship between sTREM-1 serum concentrations and admission-to-delivery interval in women with PTB.

## Materials and Methods

### Ethics Statement

The study was approved by the Ethical Committee of Ghent University hospital (EC/2009/010). All participants provided oral and written informed consent.

### Study Design and Population

We conducted a prospective cohort study at the Department of Obstetrics and Gynecology of Ghent University Hospital in which 768 pregnant women between 24 and 42 weeks’ gestation, presenting to the labor and delivery ward were enrolled, in order to build a bank of biological samples and clinical data and to explore putative associations between inflammatory markers of term and preterm labor. [24]All subjects for this study were selected from the prospective cohort except patients in group 2 (see below). A convenience sample of 176 singleton pregnancies was selected and divided into four groups according to gestational age (GA) and labor status: (1) women with preterm labor (PTL), who delivered before 34 weeks gestation (*PTB*) (n = 52). This group consisted of 35 patients with preterm premature rupture of the membranes (PPROM) and 17 with PTL and intact membranes. All patients were in labor at the time of sampling; (2) women not in labor, attending the prenatal clinic of Ghent University Hospital and matched for GA with the PTB group. All these women had an uncomplicated pregnancy that proceeded to term delivery (*GA matched controls*) (n = 52); (3) healthy pregnant women at term in labor (*AT in labor*) (n = 40). This group included patients in labor with intact membranes (n = 20) and women with premature rupture of the membranes (PROM) (n = 20). (4) healthy pregnant women at term not in labor, undergoing a primary Caesarean section *(AT not in labor*) (n = 32). Eligibility criteria included age >18 years, gestational age ≥24 weeks, absence of fetal (congenital) malformations, absence of maternal infectious disease (e.g. HIV, hepatitis B) and Dutch speaking. Data on maternal demographics, medical and obstetrical history and pregnancy outcome were recorded.

### Definitions

PTL was defined as having regular uterine contractions (six to twelve contractions in one hour) and documented cervical changes before 37 completed week’s gestation. Cervical changes include cervical effacement or dilatation, cervical shortening (<25 mm) and/or funneling and were measured by vaginal examination or transvaginal ultrasonography. PPROM was defined as amniorrhexis at least 1 h before the onset of contractions. A confirmatory test (crystallization test on slide or rapid rupture of membranes (ROM) - test (Amnisure, Boston, US)) was performed if PPROM was suspected on the basis of fluid leakage or oligohydramnion. In case of a positive test, the diagnosis of PPROM was considered. PTB was defined as PTL and/or PPROM, followed by a delivery before 34 weeks. Gestational age was determined based on last menstrual period corrected by early ultrasound before 20 weeks gestation.

#### Sample collection and processing

Blood samples of laboring women (either term or preterm) were collected by the attending midwife upon admission to the labor and delivery ward. Women at term not in labor were sampled prior to their Caesarean section. *GA matched controls* were recruited from the antenatal clinic. These pregnant women were screened at 20–22 weeks (structural ultrasound) to verify whether they fulfilled the inclusion criteria. When eligible for participationthese women were matched for week of gestation with a PTB case. Sampling was performed during a subsequent prenatal consultation at the appropriate gestational age. Samples were stored at 4°C until processing. Blood samples were centrifuged at 1000 *g* for 10 minutes at room temperature to harvest serum. All serum samples were stored at −80°C until analysis. Samples used for this study were never thawed previously. sTREM-1 concentrations were determined using an enzyme-linked immunoassay in accordance with the manufacturer’s instructions (R&D systems, Minneapolis, MN). All assay plates included samples from all groups. Both standards and samples were measured in duplicate. The interassay and intra-assay coefficients of variation were 7.8% and 13.9%, respectively.

#### Statistical analysis

Univariate group differences were tested with χ^2^ or Fisher’s Exact test and Student’s t-test or Mann-Whitney *U*-test when appropriate. The normality of the continuous data was tested using the Kolmogorov-Smirnov test and visual inspection of QQ-plots. Since the distribution of sTREM-1 was positively skewed, their natural log transformed values were used so as to have a normally distributed outcome variable for the multiple regression analysis, which was performed on the full dataset (n = 176). A backward selection procedure was applied in which covariates were sequentially removed in order of increasing significance until only terms with p-value below 0.10 remained. The subgroups were translated into three variables: preterm (vs. at term), labor (vs. not in labor) and rupture of the membranes (ROM)(vs. intact membranes). These variables are considered as key covariates and remained in the model regardless of their significance. Other covariates considered in the model selection were maternal age, educational level, marital status, smoking, body mass index (BMI), history of PTB, storage time and time delay between blood sampling and serum harvesting (further described as sample age). After backward selection of main terms, first order interactions were considered between all remaining covariates, yielding the final model. Spearman correlation was performed to estimate correlations between serum concentration of sTREM-1 and the admission-to-delivery interval in the PTB group. All statistical analyses and tests were performed two-sided at the 5% significance level using SPSS statistics 19 software (IBM, Chicago, Illinois).

## Results

### Demographic and Clinical Characteristics of the Study Population

Demographic and clinical characteristics of the study population are presented in Table 1. There were no significant differences between groups regarding pre-pregnancy BMI, marital status, ethnicity, conception, parity and history of PTB. Women with *PTB* had a significantly lower education level than *GA matched controls* (P = 0.003). Women *AT not in labor* were significantly older than women *AT in labor* (P = 0.03). There were more smokers among women *AT not in labor* and among women with *PTB* as compared to women *AT in labor* (P = 0.04 respectively P = 0.005).

**Table 1:** Demographic and clinical characteristics of the study population.

Variables	PTB (n = 52)	GA matchedcontrols (n = 52)	AT in labor(n = 40)	AT not inlabor (n = 32)	Group 1vs.2 P-value	Group3vs.4 P-value	Group 1vs.3 P-value
Maternal age (mean ± SD, y)	28.7±5.6	29.8±4.1	29.1±4.6	31.4±4.4	0.26	0.03	0.69
Pre-pregnancy BMI (Me,IQR, kg/m2)	21.5 [19.7–24.8]	21.8 [20.1–23.1]	21.9 [19.9–24.0]	21.6 [19.9–25.0]	0.98	0.43	0.77
Educational level (n, %)					0.002	0.58	0.09
Secondary education or less	24 (46.2)	9 (17.3)	11 (27.5)	7 (21.9)			
Higher education	28 (53.8)	43 (82.7)	29 (72.5)	25 (78.1)			
Marital status (n, %)					0.70	1.00	0.38
Married or cohabiting	47 (92.2)	48 (94.1)	39 (97.5)	31 (96.9)			
Living alone	4 (7.8)	3 (5.9)	1 (2.5)	1 (3.1)			
Smoking at recruitment	9 (17.3)	8 (15.4)	0 (0.0)	4 (12.5)	0.79	0.04	0.005
Ethnicity (n, %)					1.00	0.12	0.16
White/Caucasian	51 (98.1)	50 (96.2)	36 (90.0)	32 (100.0)			
Other	1 (1.9)	2 (3.8)	4 (10.0)	0 (0.0)			
GA at recruitment (Me,IQR, wk)	29.0 [26.0–31.0]	29.0 [26.0–31.0]	40.0 [39.0–40.0]	38.0 [38.0–39.0]	P = 1.00	<0.001	0.001
Conception (n, %)					0.78	0.09	0.96
Spontaneous	44 (84.6)	45 (86.5)	34 (85.0)	31 (96.9)			
Assisted reproductive technology	8 (15.4)	7 (13.5)	6 (15.0)	1 (3.1)			
Nullipara (n, %)	32 (61.5)	26 (50.0)	20 (50.0)	12 (37.5)	0.24	0.29	0.27
History of PTB	4 (7.7)	2 (3.8)	2 (5.0)	1 (3.1)	0.68	1.00	0.69
GA at delivery (Me, IQR, wk)	30.0 [28.0–32.0]	40.0 [39.0–40.0]	40.0 [39.0–40.0]	38.0 [38.0–39.0]	<0.001	<0.001	<0.001
Delivery mode (n, %)					0.21	<0.001	= 0.38
Vaginal birth	48 (92.3)	43 (84.3)	39 (97.5)	0 (0.0)			
Caesarean section	3 (5.9)	8 (15.7)	1 (2.5)	32 (100.0)			
Birth weight (mean, ±SD, g)	1517.3±514.4	3484.9±498.0	3461.9±396.2	3236.9±360.0	<0.001	0.01	<0.001
Gender (n, %)					0.04	0.75	0.04
♀	16 (30.8)	26 (51.0)	21 (52.5)	18 (56.3)			
♂	36 (69.2)	25 (49.0)	19 (47.5)	14 (43.8)			

AT, at term; BMI, body mass index; GA, gestational age; IQR, interquartile range; Me, Median; PTB, preterm birth; SD, standard deviation.

X^2^ or Fisher’s Exact test for categorical variables; Student’s t-test or Mann-Whitney *U*-test for continuous variables.

### Serum sTREM-1 Concentrations

**Figure 1:**
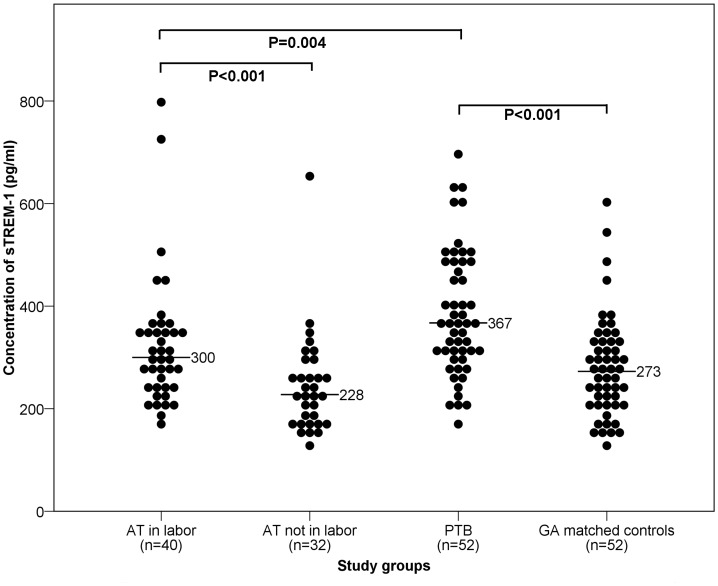
Serum sTREM-1 concentrations among groups. Median sTREM-1 concentrations are significantly elevated in women in labor (either term or preterm) vs. non-laboring controls. sTREM-1 levels are significantly higher in preterm vs. term labor. Horizontal bars denote the median value for each study group.

sTREM-1 was detected in all serum samples collected in this study. Figure 1 shows sTREM-1 concentrations among the different groups. Significantly higher median concentrations of sTREM-1 were seen in women with *PTB* compared to *GA matched controls* (367 pg/ml, interquartile range (IQR) 304–483 *vs.* 273 pg/ml, IQR 208–334; P<0.001). Median sTREM-1 concentrations were significantly increased in women *AT in labor* compared with *AT not in labor* (300 pg/ml, IQR 239–353 *vs*. 228 pg/ml, IQR 174–285; P<0.001). Women with *PTB* had significantly higher sTREM-1 levels than women *AT in labor* (367 pg/ml, IQR 304–483 *vs.* 300 pg/ml, IQR 239–353; P = 0.004) (Figure 1).

For multiple linear regression, the covariates educational level, history of PTB and sample age, met the significance criteria of the backward selection and were retained in the final model, in addition to the key covariates preterm, labor and rupture of the membranes. No interaction effects were found to be significant. Results of the final model are shown in Table 2. Since the model used the natural log of sTREM-1 concentration as the dependent variable, model coefficients reflect differences on the ln(concentration) scale. To allow interpretation on the original concentration scale, we also provide exponentiated coefficients that reflect relative (%) instead of absolute changes. The model showed that labor (vs. not in labor) and preterm (vs. not preterm), but not ROM (vs. intact membranes) remained significantly associated with sTREM-1 concentration after adjusting for educational level, history of PTB and sample age. On average, the sTREM-1 concentration was 30% higher in labor (vs. not in labor) and 15% higher preterm (vs. at term). The average sTREM-1 concentration was 14% higher in women with secondary education or less compared to women with higher education and 28% lower in women with a history of PTB versus no history. With other covariates held constant, sTREM-1 concentrations multiplied with a factor 1.004 for every additional hour of sample age.

**Table 2:** Multiple regression model for ln(sTREM-1 concentration).

Parameter	Model coefficient(95%CI)	Exponentiated coefficient (95%CI)	*P*-value
Intercept	5.416 [5.323, 5.508]	224.9 [205.1, 246.7]	<0.001
Preterm [vs. at term]	0.142 [0.043, 0.241]	1.152 [1.044, 1.272	0.005
Labor [vs. not in labor]	0.258 [0.126, 0.391]	1.295 [1.134, 1.479]	<0.001
ROM [vs. intact membranes]	−0.021 [−0.156, 0.113]	0.979 [0.856, 1.120]	0.76
Secondary education (or less) [vs. higher education]	0.128 [0.020, 0.236]	1.136 [1.020, 1.266]	0.02
History of PTB [vs. no history]	−0.324 [−0.542, −0.105]	0.724 [0.582, 0.900]	0.004
Sample age (in hours)	0.0039 [0.0003, 0.0076]	1.004 [1.000, 1.008]	0.04

Results of the model fitted on the full dataset (n = 176), obtained from the backward selection procedure outlined in the text. Covariates considered but not retained were: maternal age, marital status, smoking, body mass index and storage time. Coefficients of the model (additive on the log scale) were exponentiated to multiplicative factors, allowing interpretation on the concentration scale.

Sample age = time delay between blood sampling and processing.

CI, confidence interval; PTB, preterm birth; ROM, rupture of the membranes.

R^2^ = 0.28.

### Serum sTREM-1 Concentrations in PPROM vs. PTL and Relation with Admission-to-Delivery Interval

In the *PTB* group, no differences in sTREM-1 concentrations were observed between women with PPROM versus women with PTL and intact membranes (372 pg/ml, IQR 303–494 *vs*. 342 pg/ml, IQR 303–436; P = 0.46). This result did not change when using multiple regression analysis (data not shown). The median admission-to-delivery interval in the *PTB* group was 3,5 days (IQR 3,5–7), in women with PPROM 4 days (IQR 0-7) and in women with PTL and intact membranes 3 days (IQR 0–14,5). The concentration of sTREM-1 was not related to the admission-to-delivery interval in women with *PTB* (r = 0.17, P = 0.23) neither in the subgroups (PPROM: r = 0.30, P = 0.08; PTL and intact membranes: r = -0.11, P = 0.67).

## Discussion

We have used a case control study to assess sTREM-1 concentrations in serum during term and preterm labor. In line with previous observations in amniotic fluid [6], serum sTREM-1 levels are significantly increased in women with preterm labor compared to GA matched controls. sTREM-1 levels were also elevated in women at term in labor vs. those not in labor. Recent studies have demonstrated that sTREM-1, although initially described in microbial inflammation [8], is involved in non-infectious inflammatory conditions as well [15], [16]. There is accumulating evidence that inflammation is also important in spontaneous labor at term [3], [25]–[27]. Moreover, it has been shown that term labor is associated with an increased risk of microbial invasion of the amniotic cavity (MIAC). The more advanced the cervical dilatation, the greater the risk of MIAC [28], [29]. Our observation is consistent with Youssef et al [23] who demonstrated increased TREM-1 mRNA expression in myometrium and cervix after labor at term. In contrast, Kusanovic et al [6] found no differences in amniotic fluid concentrations of sTREM-1 between laboring and non-laboring women at term. These data suggest that the maternal inflammatory response during labor may be different from the fetal response. A large cross-sectional study is needed to evaluate sTREM-1 concentrations in both compartments during labor.

Since microbial invasion is more prevalent in PPROM [5], [30], we expected higher sTREM-1 levels in these women. Nevertheless, we found no differences in sTREM-1 concentrations between patients with PPROM and those with PTL and intact membranes. This finding may be attributed to the relative small number of patients in both groups. In the presence of intra-amniotic infection, sTREM-1 levels in amniotic fluid were higher in women with PPROM vs. PTL and intact membranes [6]. This observation suggests that sTREM-1 is probably a good marker for intra-amniotic infection in amniotic fluid but not in maternal serum which has been recently demonstrated by Cobo et al [21]. They evaluated 27 proteins in maternal serum of women with PPROM or PTL and intact membranes and observed a weak maternal inflammatory response in women with MIAC. In particular, serum TREM-1 levels did not differ between women with and without MIAC. Moreover, differences in protein levels were only evident at early gestational age (less than 32 weeks of gestation). Similar observations were made in amniotic fluid of women with PPROM. TREM-1 concentrations did not differ between women with and without MIAC [20], but higher levels were observed in PPROM cases <34 weeks in the presence of both MIAC and histological chorioamnionitis [31]. This indicates that TREM-1 may serve as a good marker for severe inflammation in a subset of pregnant women at risk for PTB.

In addition, we observed significantly higher sTREM-1 levels in preterm labor compared to term labor. The fact that microbial invasion is more common in preterm birth could explain this result. Another explanation could be that sTREM-1 levels alter during pregnancy and may differ from the baseline in these women. However, our study was not designed to evaluate longitudinal changes in sTREM-1 concentrations. A study in which sTREM-1 levels are serially assayed throughout gestation and in non-pregnant women would be able to address this issue.

It is also recommendable to evaluate whether sTREM-1 levels differ between women with PTL and intact membranes who delivered preterm and those who delivered at term. We carried out a preliminary evaluation, but found no significant differences in sTREM-1 levels between both groups (data not shown). This result must be interpreted cautiously since the number of patients with PTL who delivered at term was rather low (n = 10). However, Tsiartas et al [22] did not observed higher levels of TREM-1 in women with PTL who delivered within 7 days vs. those delivering later.

Variability in pre-analytical factors has been shown to influence cytokine levels. Cytokine concentrations are most critically affected by sample age i.e. the time lapse between blood collection and processing [32]–[35]. The window between collection and processing and the variability between samples has to be minimized, but is not always feasible in practice [32], [33]. The impact of sample age on levels of inflammatory markers is often poorly addressed in studies. Our model suggests that sample age can affect sTREM-1 measurements in serum, supporting the need to standardize specimen processing as much as possible and/or to consider differences due to sample age.

Some limitations of this study deserve consideration. First, the case control study design did not allow investigating the value of serum sTREM-1 to predict the onset of PTB. Previous studies found that increased sTREM-1 levels in the second trimester were associated with PTB in asymptomatic high risk patients, but not in low risk women [17], [19]. Further research is needed to establish the value of sTREM-1 as a predictive marker of PTB. Second, no information could be obtained on the presence of intra amniotic infection in patients with PPROM or PTL and sTREM-1 levels in serum and amniotic fluid could not be compared, since amniocentesis is not routinely performed in patients with preterm labor. Finally, sTREM-1 concentrations were measured only once upon admission. Evaluation of the time-course of plasma sTREM-1 levels during sepsis showed that a progressive decline in sTREM-1 concentration was associated with a favorable clinical evolution [36]. It would be interesting to investigate the clinical informative value of repeated determinations of serum sTREM-1 in hospitalized patients with preterm labor.

In conclusion, we found elevated sTREM-1 concentrations in maternal serum during spontaneous parturition (either term or preterm) and sTREM-1 levels were significantly higher in women with preterm labor. Further studies are required to explore the role of sTREM-1 in the inflammatory response during pregnancy and labor.
